# Case Report: Neoadjuvant and Adjuvant Crizotinib Targeted Therapy in Stage IIIA-N2 ALK-Positive Non-Small-Cell Lung Cancer

**DOI:** 10.3389/fonc.2021.655856

**Published:** 2021-03-17

**Authors:** Xiao-Hong Xie, Ze-Jiang Zhan, Yin-Yin Qin, Ju-Hong Jiang, Wei-Qiang Yin, Rong-Hui Zheng, Shi-Yue Li, Cheng-Zhi Zhou

**Affiliations:** ^1^ Department of Pulmonary and Critical Care Medicine, The First Affiliated Hospital of Guangzhou Medical University, Guangzhou Institute of Respiratory Health, State Key Laboratory of Respiratory Disease, National Clinical Research Center for Respiratory Disease, Guangzhou, China; ^2^ Guangzhou Medical University, Guangzhou, China; ^3^ Department of Radiation Oncology, The Affiliated Cancer Hospital & Institute of Guangzhou Medical University, Guangzhou, China; ^4^ Department of Respiratory Pathology, The First Affiliated Hospital of Guangzhou Medical University, Guangzhou Institute of Respiratory Health, State Key Laboratory of Respiratory Disease, National Clinical Research Center for Respiratory Disease, Guangzhou, China; ^5^ Department of Thoracic Surgery and Oncology, The First Affiliated Hospital of Guangzhou Medical University, Guangzhou Institute of Respiratory Health, State Key Laboratory of Respiratory Disease, National Clinical Research Center for Respiratory Disease, Guangzhou, China

**Keywords:** locally advanced non-small-cell lung cancer, ALK inhibitor, neoadjuvant, adjuvant, targeted therapy

## Abstract

The treatment of anaplastic lymphoma kinase (ALK)-positive locally advanced non-small-cell lung cancer (NSCLC) is challenging because there is no randomized controlled trial has been reported. The value of neoadjuvant and adjuvant targeted therapy remains unclear. Herein, we show that systemic treatment with ALK inhibitor crizotinib before surgery can provide the potential to cure the initially inoperable tumor. A 27-year-old man was diagnosed with a stage IIIAcT3N2M0 (7^th^UICC/AJCC) upper left lung adenocarcinoma harboring EML4-ALK fusion gene. Clinically, the patient had a large primary lesion adjacent to the pericardium and regional lymph node metastasis at the ipsilateral mediastinum. Poor tumor response was observed after 3 cycles of chemotherapy (gemcitabine plus cisplatin), and upon multidisciplinary discussion, the patient was started with 250 mg crizotinib twice daily. Successive clinical examinations showed a progressive reduction of the lesions. After 2 months of therapy, the patient was downstaged to cT2aN2M0, then video-assisted thoracic surgery was performed and the final histopathological stage was ypT2aN2M0. The treatment with crizotinib (250 mg, qd) was continued more than 30 months post surgery and stopped until intracranial oligometastasis. The patient’s overall survival (OS) time is 68 months at last follow-up. This case presented here supports the use of neoadjuvant and adjuvant treatment with ALK inhibitors in ALK positive locally advanced NSCLC.

## Introduction

According to the report of the International Agency for Research on Cancer, 2,093,876 cases of lung cancer were newly diagnosed worldwide in 2018, accounting for 11.6% of newly diagnosed cancers in the same year (1). Approximately 85% of lung cancers are classified as non-small-cell lung cancer (NSCLC), and 30% of which are defined as stage III at diagnosis (2, 3).

Stage III NSCLC is considered highly heterogeneous. Most patients have lost the opportunity for radical surgery at the time of initial diagnosis. The overall clinical efficacy of treatment for stage III NSCLC is not satisfactory, with 5-year survival rates of only 13~36% despite multimodality treatments (4, 5). In recent years, as small-molecule tyrosine kinase inhibitors (TKIs) have been widely used in patients with advanced NSCLC with genetic mutations, the survival outcomes of patients have become highly promising, and the benefits are obvious (6–9), reflecting the need to explore the application of small-molecule TKIs in locally advanced NSCLC with genetic mutations.

For locally advanced NSCLC with epidermal growth factor receptor (EGFR) mutation, previous studies (10–12) have focused on the application of neoadjuvant and adjuvant targeted therapy, and the results are promising. Among these patients, neoadjuvant targeted therapy is significantly superior to chemotherapy, and the regression of the primary tumor is more pronounced, rendering patients more likely to be eligible for radical surgery (10–12). Adjuvant targeted therapy offers a convenient treatment option, and patients can also benefit from survival outcomes (10). However, no randomized controlled trial for patients with anaplastic lymphoma kinase (ALK)-positive locally advanced NSCLC has been reported. The value of neoadjuvant and adjuvant targeted therapy remains unclear and requires further exploration. This case report describes initial crizotinib use as neoadjuvant and adjuvant targeted therapy for an ALK-positive locally advanced NSCLC patient.

## Case Presentation

In April 2015, a 27-year-old male who presented with cough and phlegm was diagnosed with left upper lung adenocarcinoma by biopsy, with a performance status score of 1. The histological features were characterized by solid growth with mucus production (60%) and acinar growth (40%). The patient’s clinical stage was cT3N2M0 (stage IIIA, 7^th^ UICC/AJCC). After preoperative evaluation, the initial tumor was considered unresectable since it was adjacent to the pericardium, and regional lymph node metastasis at the ipsilateral mediastinum was observed.

**Figure 1:**
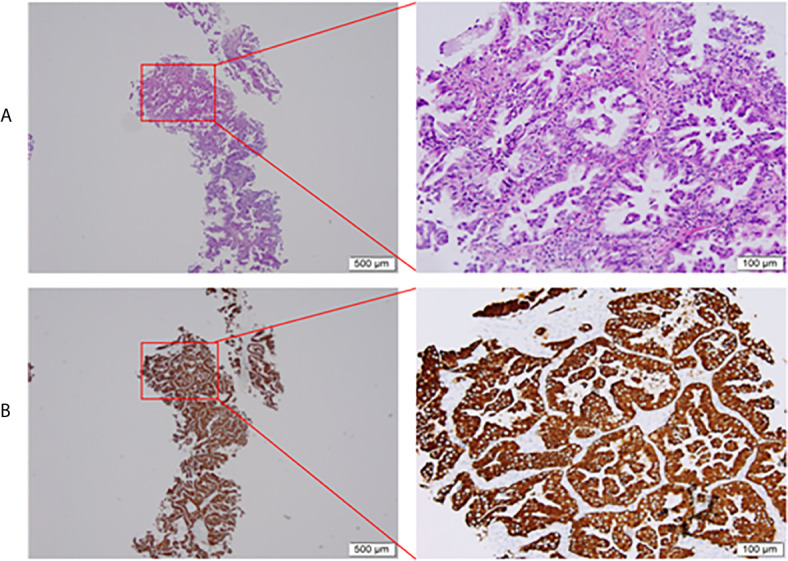
Pretreatment biopsy. **(A)** Histological aspect of lung adenocarcinoma (HE staining), **(B)** Intense cytoplasmic ALK protein expression on immunohistochemistry. HE, hematoxylin-eosin; ALK, anaplastic lymphoma kinase.

**Figure 2:**
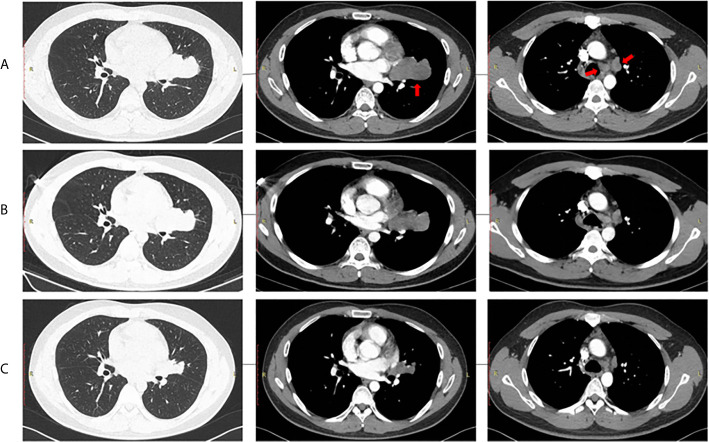
Radiological evaluation of the primary tumor and lymph nodes. **(A)** Baseline, **(B)** After 3 chemotherapy cycles, **(C)** After 2 months of crizotinib treatment.

Three cycles of gemcitabine (1250mg/m^2^, day 1 and day 8) plus cisplatin (75 mg/m^2^, day 1) were given after diagnosis. Simultaneously, the positive ALK expression was detected by Ventana immunohistochemistry (IHC) staining (**Figure 1**). Contrast chest computed tomography (CT) after 3 cycles of gemcitabine plus cisplatin showed that the primary tumor remained stable according to the RECIST 1.1 criteria compared with the initial CT scan (**Figures 2A, B**). Thereafter, crizotinib (250 mg, bid) was adopted, replacing the initial treatment. Encouragingly, the patient was downstaged to cT2aN2M0 within 2 months (**Figure 2C**).

**Figure 3:**
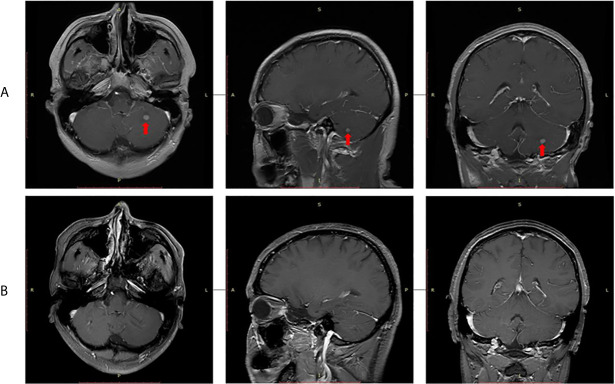
Radiological evaluation of intracranial oligometastasis. **(A)** Initial detection, **(B)** Five months after stereotactic radiosurgery.

In September 2015, video-assisted thoracic surgery (VATS) with left upper lobectomy plus lymph node dissection and partial pericardial resection was performed. The pathological stage was ypT2aN2M0. Then, postoperative radiotherapy (PORT) was performed with a total dose of 50 Gy in 25 fractions. Given the limitation of IHC detection, genomic sequencing was performed for the patient’s resected tumor tissue sample. Next-generation sequencing (NGS) analysis was carried out using a commercially available capture-based targeted sequencing panel, targeting 8 genes (ALK, BRAF, EGFR, ERBB2, KRAS, MET, RET and ROS1) (Burning Rock Biotech, Guangzhou, China). EML4-ALK (E13:A20) fusion oncogene was found by NGS assay. Crizotinib (250 mg, qd) was administered as adjuvant targeted therapy until an intracranial oligometastasis was first detected in July 2018, with a first progression-free survival (PFS 1) time of 39 months. Subsequently, stereotactic radiosurgery (SRS) of the intracranial oligometastasis followed by crizotinib treatment (250 mg, bid) was performed. Five months after SRS, the intracranial oligometastasis showed a complete response based on the RECIST 1.1 criteria (**Figure 3**). At the final follow-up in December 2020, no grade 3/4 adverse events or disease progression had occurred. Remarkably, a second PFS time has not been reached. The patient’s overall survival (OS) time is currently 68 months.

## Discussion

Based on NCCN guidelines, stage III NSCLC patients should receive multimodality therapies. Radical resection is recommended for operable patients. Potentially operable patients can gain opportunities for radical resection through neoadjuvant therapies including chemotherapy, radiotherapy, or a combination of both. Additionally, concurrent radical chemoradiotherapy followed by immunotherapy maintenance is preferred for inoperable patients. Notably, for locally advanced NSCLC patients with gene mutations, the NCCN guidelines do not indicate whether targeted therapy is superior to traditional chemotherapy as neoadjuvant or adjuvant treatment. This case report describes the application of neoadjuvant and adjuvant targeted therapy in a patient with stage IIIA-N2 ALK-positive NSCLC for the first time.

Approximately 7% of NSCLCs have ALK gene fusions, and EML4 is the most common ALK fusion partner, accounting for 90~95% of ALK rearrangements (13). A variety of rare ALK fusion partners, such as KIF5B, KLC1, TFG, TPR, HIP1, STRN, DCTN1, SQSTM1, NPM1, BCL11A and BIRC6, have also been detected, summarized in a study by Du Xue et al. and colleagues (14). EML4-ALK is further divided into diverse fusion subtypes, among which V1 (E13:A20) and V3 (E6:A20) account for the highest proportion, both at approximately 32%, and other EML4-ALK fusion subtypes are relatively rare (each accounts for less than 10%) (13). The identification of these mutations enables target-specific therapies. The application of ALK inhibitors indeed prolongs patient survival; however, a certain proportion of patients will inevitably acquire resistance to ALK inhibitors (15, 16). Therefore, it is of great significance to identify predictors of response or resistance to ALK inhibitors. In addition, NGS of dynamic plasma or tissue gene mutation abundance detection is effective in predicting the response to ALK inhibitors, as illustrated by Zhang Chao et al. (17). Hence, future clinical research in the era of precision targeted therapy should take dynamic NGS into account.

Crizotinib is an oral small-molecule TKI of ALK, MET, and ROS1 kinases (18) and is a Category 1 recommendation for advanced ALK-positive NSCLC according to the NCCN guidelines since previous studies have demonstrated its superior efficacy over chemotherapy (9, 19). In a profile 1014 study (9), crizotinib was associated with better progression-free survival (PFS) compared to pemetrexed plus cisplatin or carboplatin chemotherapy in patients with advanced ALK-positive NSCLC (median, 10.9 months *vs.* 7.0 months; P<0.001). A similar outcome was also observed in a profile 1029 study (20). Thus, the potential clinical benefits of targeted therapy adoption for ALK-positive locally advanced NSCLC should also be explored. In this case, the patient was not sensitive to first-line chemotherapy with gemcitabine plus cisplatin and was therefore switched to crizotinib based on his gene mutations, which enabled radical surgery. Kaoru Kaseda et al. and colleagues (21) reported the first case of crizotinib application before surgical resection in ALK-positive NSCLC and showed promising disease reduction. Zhang et al. showed that neoadjuvant crizotinib can achieve complete resection (22). Moreover, the SAKULA trial demonstrated that ceritinib contributed to a high pathologic response rate in ALK-positive resectable locally advanced NSCLC (23). Dumont and colleagues (24) explored crizotinib as a second-line neoadjuvant treatment when chemotherapy failed to achieve satisfactory regression of the tumor burden and observed no disease recurrence at 18 months post-surgery. However, whether the benefit of neoadjuvant ALK inhibitors can transform into long-term survival is controversial. In the present case, unlike the studies of Kaoru Kaseda and Delphine Dumont, a long-term follow-up of 68 months was performed (21, 24).

Previous studies (25, 26) have demonstrated that stage N2 NSCLC patients benefit from PORT. However, in the CTONG 1103 trial (10), PORT was not administered. Nevertheless, PORT was adopted for this patient. In terms of adjuvant targeted therapy, earlier trials (11, 12) explored the application of oral TKIs for EGFR-positive NSCLC patients, and the results were encouraging. ADJUVANT (11) is the first prospective study comparing gefitinib with vinorelbine plus cisplatin as adjuvant treatments in patients with EGFR-positive stage II-IIIA (N1-N2) NSCLC, which showed that gefitinib significantly prolongs PFS (median, 28.7 months *vs.*18.0 months; P = 0.0054). The EVAN study (12) explored erlotinib *vs.* vinorelbine plus cisplatin as adjuvant treatments in patients with EGFR-positive stage IIIA NSCLC and demonstrated substantially higher 2-year PFS rates of 81.2% in the erlotinib group and 44.6% in the chemotherapy group with oral TKI administration (P<0.001), reflecting the reliability of adopting oral TKIs as adjuvant treatment for certain groups. No relevant study has described ALK-positive NSCLC patients in this setting. Ultimately, the patient was given adjuvant crizotinib targeted therapy and has achieved considerable efficacy in terms of both PFS and OS, with a PFS 1 of 39 months and an OS of 68 months thus far. Notably, clinical stage IIIB NSCLC is associated with a poor survival outcome, with a median survival time of approximately 14.1 months (5), which is substantially shorter than the PFS 1 of 39 months in this case.

To date, this is the first case of stage IIIA-N2 ALK-positive NSCLC treated with neoadjuvant and adjuvant targeted therapy combined with surgery and PORT to show remarkable clinical efficacy. Further exploration of this treatment model for stage III ALK-positive NSCLC is urgently needed.

## Data Availability Statement

The original contributions presented in the study are included in the article/supplementary material. Further inquiries can be directed to the corresponding author.

## Ethics Statement

Ethical review and approval was not required for the study on human participants in accordance with the local legislation and institutional requirements. The patients/participants provided their written informed consent to participate in this study. Written informed consent was obtained from the individual(s) for the publication of any potentially identifiable images or data included in this article.

## Author Contributions

1. Guarantor of integrity of the entire study: C-ZZ. 2. Study concepts and design: Y-YQ, J-HJ. 3 Literature research: W-QY, R-HZ, S-YL. 4. Manuscript preparation: X-HX, Z-JZ. 5. Manuscript editing: C-ZZ. All authors contributed to the article and approved the submitted version.

## Funding

This report was supported by grants from the State Key Laboratory of Respiratory Disease- The open project [grant number: SKLRD-OP-2018011], the State Key Laboratory of Respiratory Disease- The Independent project [grant number: SKLRD-QN-201720], Wu Jieping Fund-Ministry of Health Project [grant number: 320.6750.18125], Guangdong High Level University Clinical Cultivation Project [grant number: 2017-21020].

## Conflict of Interest

The authors declare that the research was conducted in the absence of any commercial or financial relationships that could be construed as a potential conflict of interest.
